# Single-molecule characterization of salivary protein aggregates from Parkinson’s disease patients: a pilot study

**DOI:** 10.1093/braincomms/fcae178

**Published:** 2024-05-21

**Authors:** Martin Furlepa, Yu P Zhang, Evgeniia Lobanova, Lakmini Kahanawita, Giorgio Vivacqua, Caroline H Williams-Gray, David Klenerman

**Affiliations:** Department of Chemistry, University of Cambridge, Cambridge CB2 1EW, UK; Department of Clinical Neurosciences, University of Cambridge, Cambridge CB2 0PY, UK; Department of Chemistry, University of Cambridge, Cambridge CB2 1EW, UK; UK Dementia Research Institute at Cambridge, Cambridge CB2 0XY, UK; Department of Chemistry, University of Cambridge, Cambridge CB2 1EW, UK; UK Dementia Research Institute at Cambridge, Cambridge CB2 0XY, UK; Department of Clinical Neurosciences, University of Cambridge, Cambridge CB2 0PY, UK; Microscopic and Ultrastructural Anatomy Research Unit-Integrated Research Centre (PRABB), Campus Biomedico University of Rome, 00128 Rome, Italy; Department of Clinical Neurosciences, University of Cambridge, Cambridge CB2 0AH, UK; Department of Clinical Neurosciences, University of Cambridge, Cambridge CB2 0PY, UK; Department of Chemistry, University of Cambridge, Cambridge CB2 1EW, UK; UK Dementia Research Institute at Cambridge, Cambridge CB2 0XY, UK

**Keywords:** Parkinson’s disease, single-molecule imaging, α-synuclein, amyloid-β, saliva

## Abstract

Saliva is a convenient and accessible biofluid that has potential as a future diagnostic tool for Parkinson’s disease. Candidate diagnostic tests for Parkinson’s disease to date have predominantly focused on measurements of α-synuclein in CSF, but there is a need for accurate tests utilizing more easily accessible sample types. Prior studies utilizing saliva have used bulk measurements of salivary α-synuclein to provide diagnostic insight. Aggregate structure may influence the contribution of α-synuclein to disease pathology. Single-molecule approaches can characterize the structure of individual aggregates present in the biofluid and may, therefore, provide greater insight than bulk measurements. We have employed an antibody-based single-molecule pulldown assay to quantify salivary α-synuclein and amyloid-β peptide aggregate numbers and subsequently super-resolved captured aggregates using direct Stochastic Optical Reconstruction Microscopy to describe their morphological features. We show that the salivary α-synuclein aggregate/amyloid-β aggregate ratio is increased almost 2-fold in patients with Parkinson’s disease (*n* = 20) compared with controls (*n* = 20, *P* < 0.05). Morphological information also provides insight, with saliva from patients with Parkinson’s disease containing a greater proportion of larger and more fibrillar amyloid-β aggregates than control saliva (*P* < 0.05). Furthermore, the combination of count and morphology data provides greater diagnostic value than either measure alone, distinguishing between patients with Parkinson’s disease (*n* = 17) and controls (*n* = 18) with a high degree of accuracy (area under the curve = 0.87, *P* < 0.001) and a larger dynamic range. We, therefore, demonstrate for the first time the application of highly sensitive single-molecule imaging techniques to saliva. In addition, we show that aggregates present within saliva retain relevant structural information, further expanding the potential utility of saliva-based diagnostic methods.

## Teaser

Using a single-molecule approach, Furlepa *et al*. find an increased α-synuclein-to-amyloid-β aggregate ratio in the saliva of patients with Parkinson’s disease. Using super-resolution microscopy, they find Parkinson’s disease saliva to contain morphologically distinct amyloid-β aggregates. Combining these methods allows patients with Parkinson’s disease to be distinguished from controls.

## Graphical Abstract

**Graphical Abstract:**
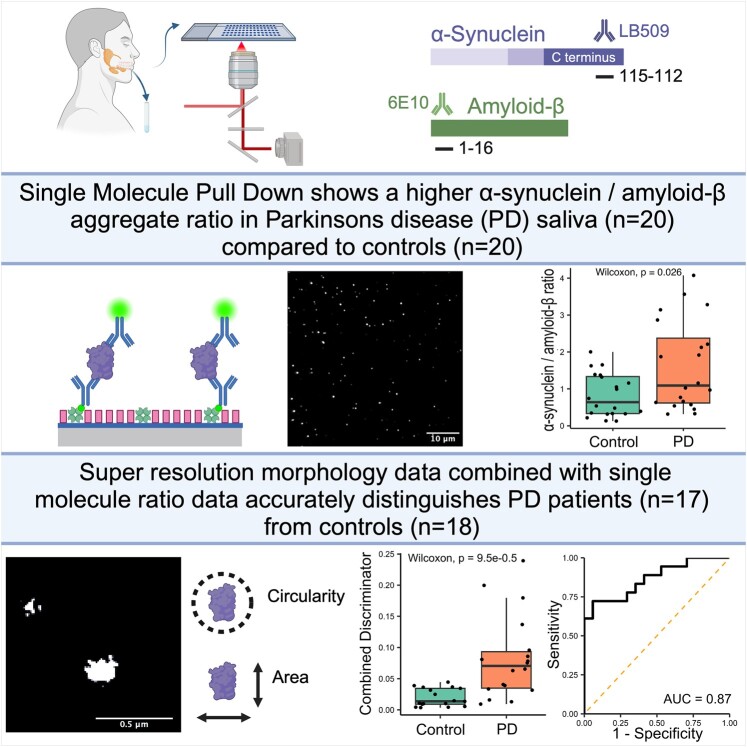

## Introduction

Parkinson’s disease is the second most common neurodegenerative disorder affecting 1% of those over 60 years of age.^1^ Classically, Parkinson’s disease is characterized by its motor features of bradykinesia, rigidity and resting tremor, although non-motor manifestations are now more widely appreciated and incorporated as supportive features in diagnostic criteria.^2^ Pathologically, the disease is characterized by the accumulation of intracellular Lewy bodies within neurons. α-Synuclein is a key component of Lewy bodies,^3,4^ and a substantial body of evidence implicates abnormal aggregation of α-synuclein and toxic oligomeric species as a key driver of disease progression.^5^ Further, mutations of the gene encoding α-synuclein lead to autosomal dominant forms of Parkinson’s disease, further implicating its abnormal aggregation in disease progression.^6,7^

The diagnosis of Parkinson’s disease primarily relies on clinical assessment, which has a diagnostic accuracy of ∼80% in patients with symptoms onset >5 years,^8^ with lower accuracy earlier in the disease course.^9^ To date, there is no single biomarker that can accurately diagnose Parkinson’s disease in clinical practice. Current treatment options focus on relieving Parkinson’s disease symptoms by stimulating the dopamine system, but they do not slow or prevent disease progression. Novel therapies, which utilize targeted antibodies to remove α-synuclein aggregates from neuronal tissue, have failed to alter the disease trajectory.^10,11^ The reliance on clinical diagnosis, which typically occurs after motor symptoms develop, may impede the development of new therapies, as neuronal tissue loss within the substantia nigra precedes motor manifestations.^12^ An accurate, easily accessible biomarker that could facilitate early diagnosis and longitudinal sampling could aid the development and utilization of new therapies.

Outside of the clinical setting, seed amplification assays (SAAs) have been shown to accurately diagnose patients based on α-synuclein seeding activity using CSF samples.^13^ However, CSF is not easily obtainable and requires the patient to undergo a minor procedure for sample collection. A new method combining SAA and immunoprecipitation has recently reported the detection of α-synuclein seeds within serum and applied this to accurately identify patients with Parkinson’s disease,^14^ raising the possibility that this highly accurate method could be applied to a more easily accessible biofluid. An alternative blood-based approach, which isolated CNS α-synuclein from blood exosomes, has reported a high degree of accuracy when distinguishing between patients with Parkinson’s disease, patients with multiple system atrophy and healthy controls.^15,16^

Saliva represents a potential alternative biofluid to blood or CSF with several benefits. Sample collection is painless for patients, staff do not require specific training for collection and there is potential for patients to collect their own samples. Previous studies evaluating saliva for the diagnosis of Parkinson’s disease have had mixed results (reviewed in Goldoni *et al*.^17^). Studies utilizing western blot, enzyme-linked immunosorbent assay (ELISA) or Luminex assays to measure total α-synuclein have found reduced levels in Parkinson’s disease saliva^18-20^ or have been unable to detect any difference.^21-25^ Other studies that used ELISA to measure oligomeric α-synuclein levels have shown that there is a greater concentration in Parkinson’s disease saliva^19,22,25,26^ and that the oligomeric to total α-synuclein ratio is raised in Parkinson’s disease, providing greater discrimination between groups than either measure alone. Comparisons of salivary α-synuclein have largely been unable to distinguish between Parkinson’s disease stages, although one study has shown a correlation between total α-synuclein concentration and Parkinson’s disease severity.^19^ More recently, SAAs have been applied to saliva showing greater seeding activity within the saliva of patients with Parkinson’s disease in comparison with controls.^27,28^ Overall, these studies suggest that α-synuclein can be reliably detected in saliva and that the concentration of different species of α-synuclein aggregates may change between health and disease.

Previous studies investigating saliva as a biofluid have used methods that provide bulk measurements of protein concentration but do not provide insight into the size or structure of different species of aggregates present within this heterogeneous population. Across a range of neurodegenerative diseases, it has been shown that aggregate structure is a key determinant of their toxicity^29^ and that in Parkinson’s disease, small soluble oligomeric forms of α-synuclein may contribute to disease progression.^5,30^ Single-molecule approaches can be used to provide a count of the number of individual protein aggregates, providing insight into their structure. This can then be combined with super-resolution microscopy to visualize individual aggregates to a 20 nm spatial resolution, providing detailed morphological information.

It has recently been shown that an aptamer-based single-molecule pulldown (SiMPull) assay can accurately distinguish between Parkinson’s disease and healthy control serum samples by measuring an increase in the ratio of α-synuclein/Aβ aggregates in patients with Parkinson’s disease.^31^ This measure, when combined with super-resolved morphology data, provides superior group discrimination. This confirmed earlier work that utilized immunodepletion in combination with Aptamer DNA Points Accumulation for Imaging in Nanoscale Topography,^32^ a related single-molecule technique, which also showed an increased α-synuclein/amyloid-β (Aβ) ratio in patients with Parkinson’s disease. Previous work has identified Aβ co-pathology in patients with Parkinson’s disease. Studies have shown that CSF Aβ_42_ decreases with worsening cognition in Parkinson’s disease^33^ and that this change is detectable several years prior to the onset of detectable cognitive decline.^34^ In addition, Aβ may act synergistically to increase the aggregation of α-synuclein in Parkinson’s disease.^35^ Concurrent measurement of both α-synuclein and Aβ provides a means of adjusting for variation in total aggregate count between participants, while also capturing additional information related to Aβ co-pathology.

In this study, we use SiMPull to capture soluble α-synuclein and Aβ aggregates in the saliva of patients with Parkinson’s disease and controls.^36^ We implement this single-molecule imaging technique using paired capture and detection antibodies targeting the same epitope to detect aggregates of interest, while excluding monomeric forms. We subsequently apply direct Stochastic Optical Reconstruction Microscopy (dSTORM) to super-resolve the captured aggregates,^37^ providing information regarding their size and morphology alongside their single-molecule count to investigate whether these methods in combination can provide greater diagnostic insight than more conventional methods.

## Materials and methods

### Participants

Ethical approval for the study was granted by the NRES Committee East of England—Central Cambridge (03/303). All participants provided written consent. The study was conducted in accordance with the Declaration of Helsinki. Patients diagnosed with idiopathic Parkinson’s disease by a movement disorder specialist according to the UK Parkinson’s Disease Brain Bank criteria^38^ were recruited from the Parkinson’s Disease Research Clinic at the John Van Geest Centre for Brain Repair, University of Cambridge, UK. Healthy, age-matched controls were recruited from the same centre and were often the partners or relatives of patients attending the clinic. Demographic data, medical history and medication use were collected from all participants. In addition, patients with Parkinson’s disease completed standardized assessments of disease severity, including the Movement Disorder Society’s Unified Parkinson’s Disease Rating Scale (MDS-UPDRS) and Hoehn and Yahr scale, and neuropsychological tests including the Montreal Cognitive Assessment (MoCA; Table 1). Levodopa equivalent daily dose was calculated using the conversion factors detailed in the systematic review by Tomlinson *et al*.^39^

**Table 1:** Summary of patient demographic and clinical characteristics for Subgroups 1 and 2

	Subgroup 1			Subgroup 2		
	PD	Control	*P*-value	PD	Control	*P*-value
Sample size	10	10		10	10	
Age	67.04 ± 5.35	74.15 ± 7.90	0.03	62.23 ± 2.46	69.37 ± 7.85	0.065
% Female	40%	50%	0.739	40%	70%	0.366
Disease duration (years)	1.10 ± 1.54			2.75 ± 2.46		
MoCA score	26.80 ± 1.87			25.5 ± 3.24		
Number of patients satisfying criteria for Parkinson’s disease MCI (MoCA <26)	2			4		
MDS-UPDRS Total	43.7 ± 15.56			54.5 ± 20.25		
MDS-UPDRS-III	28 ± 10.75			29.90 ± 12.20		
L-DOPA equivalent dose	290 ± 301.66			324.10 ± 145.16		
Hoehn and Yahr ≤2	90%			90%		
Hoehn and Yahr =3	10%			10%		

### Saliva collection and processing

Participants were assessed for eligibility to provide a saliva sample. All participants were required to fast for at least 1 h prior to sample collection. Exclusion criteria included patient reported ongoing dental or oral disease, smoking within 4 h of sample collection and consuming alcohol within 12 h of sample collection. Samples were collected between 10 am and 12 pm using the passive drool method. Participants allowed saliva to pool in their mouth and drooled into a sterile container for 5 min; typically, 1–4 ml of saliva was collected per participant. No stimulation was applied. All samples were collected and processed over ice. After collection, protease inhibitors (Sigma P2714, ×10 concentration, 10 µl/ml of saliva) and a phosphatase inhibitor (sodium orthovanadate 3 µl/ml of saliva) were added to preserve the aggregates’ structure. Samples were then centrifuged at 2600*×g* at 4°C for 15 min. The supernatant was collected and underwent a further 15 000*×g* centrifuge step at 4°C for 15 min. The resulting supernatant was then removed and stored in 100 µl aliquots in cryotubes at −80°C. Received 100 µl aliquots were then defrosted and divided into smaller 11 µl aliquots before being stored at −80°C for further analysis. There were no further freeze–thaw cycles.

### Materials and antibody preparation

Phosphate-buffered saline (PBS; Gibco PBS pH 7.4 1×, Cat. 1001023) was filtered before use (Whatman Anotop 25/0.02, Cytiva, 6809-2002). Biotinylated azide-free LB509 antibodies (Biolegend, Cat. 807710) were used to capture α-synuclein aggregates. Biotinylated azide-free 6E10 antibodies (Biolegend, Cat. 803007) were used as the capture antibody for Aβ-containing aggregates. All capture antibodies were diluted to 10 nM concentration in PBS.

α-Synuclein aggregates were detected using Alexa 647–labelled LB509 antibodies (Santa Cruz, sc-58480, 647). For the detection of Aβ aggregates, Alexa 647–labelled 6E10 antibodies (Biolegend, Cat. 803021) were used. Detection antibodies were diluted to 0.5 and 5 nM concentrations, respectively, in PBS.

Prior to SiMPull experiments, saliva samples were defrosted and diluted 10-fold in PBS. All sample preparation was completed on ice.

Total aggregate concentrations were measured as duplicates using Invitrogen ELISA kits, according to the manufacturer’s instructions (Thermo Fisher, α-synuclein #KBH0061, Aβ_40_ #KBH3481 and Aβ_42_ #KBH3441).

### Slide preparation

Slide preparation and passivation were completed as previously described using F-127 passivation (Y. P. Zhang *et al.*, submitted for publication). In brief, glass coverslips (26 × 76 mm, thickness #1.5, VWR, Cat. MENZBC026076AC40) were cleaned for 15 min in an argon plasma cleaner. After cleaning, a 50-well Polydimethylsiloxane chamber gasket (GRACE Bio-Labs CultureWell 50—3 mm diameter × 1 mm depth, Cat. 103350) was applied to the coverslip. Each well was then filled with 6 µl of a 1:1 mixture of Rain-X (Rain-X Rain Repellent) and isopropanol which was allowed to dry with the residue coating the glass surface. The RainX–isopropanol mixture was filtered (Millex, SLGV004SL) prior to use. Each well was then washed twice with PBS, and 10 µl of NeutrAvidin 0.1 mg/ml (Thermo Scientific, 31000) diluted using PBS was applied and incubated for 10 min before removal and a further two washing steps with PBS. Passivation was then completed in each well with the addition of 10 µl of F-127 (Invitrogen, P6866, 1% diluted in PBS, filtered 0.22 µm) which was incubated for 45 min before removal and a further two washing steps with PBS-T (0.05% Tween 20, Fisher BioReagents™ in 1X PBS, Gibco™).

### SiMPull assay and dSTORM protocol

**Figure 1:**
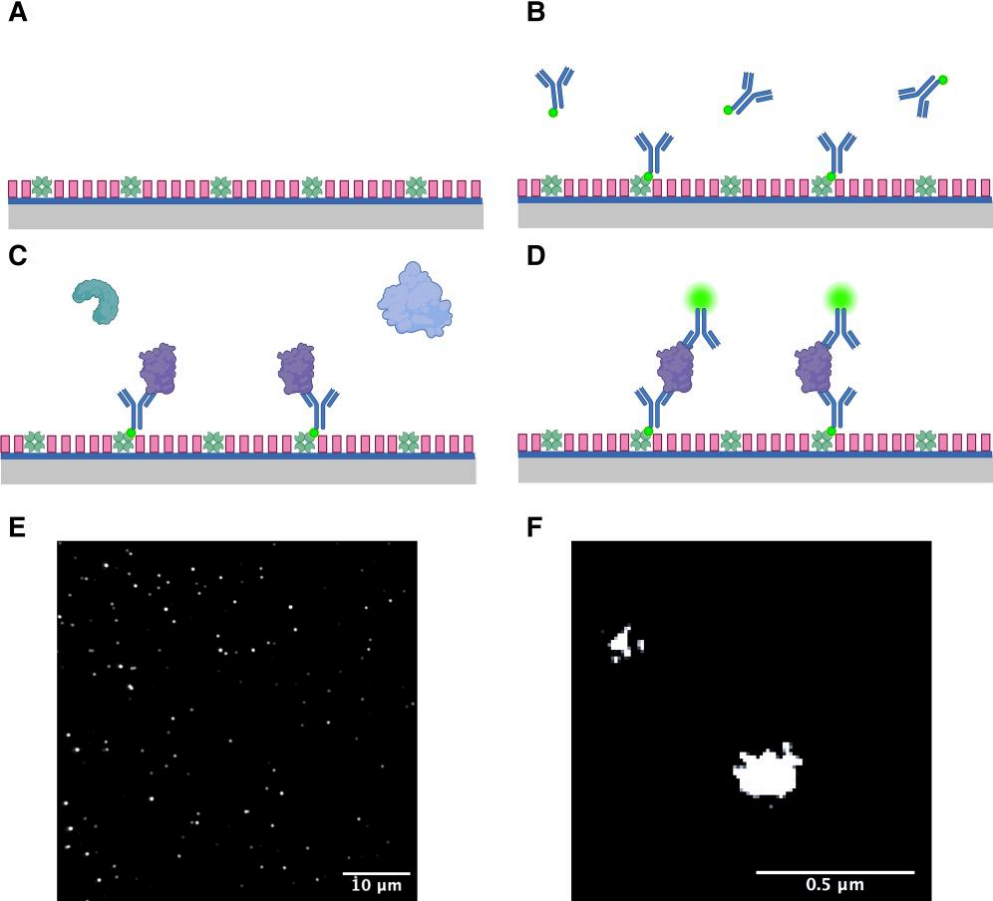
**Illustration of the SiMPull method.** (**A**) Prepared glass coverslip with NeutrAvidin bound to its surface and F-127 passivation. (**B**) Application of biotinylated capture antibodies, either LB509 or 6E10 antibody is used for the detection of α-synuclein- or Aβ-containing aggregates, respectively. (**C**) Application of saliva biofluid to the coverslip; Aβ- or α-synuclein-containing aggregates are specifically captured by the relevant immobilized antibodies on the coverslip surface. (**D**) Alexa-647-labelled LB509 and 6E10 antibodies are applied to the surface for aggregate detection. (**E**) Representative diffraction-limited image captured using Total Internal Reflection Fluorescence (TIRF) microscope set-up. (**F**) Representative super-resolved aggregates.

Ten microlitres of capture antibody were added to each well and incubated for 5 min before removal (Fig. 1), each well was then washed twice with PBS-T. Ten microlitres of saliva were added to each well and incubated for 90 min before removal and a further two PBS-T washing steps. Sample group was not blinded to the investigator. Ten microlitres of detection antibody were added to each well and allowed to incubate for 30 min. Detection antibodies were then removed, and each well was washed twice with PBS-T before being filled with 10 µl PBS to prevent the slide from drying out during image acquisition. The same antibody was used for detection and capture.

Diffraction-limited imaging was performed first and then STORM buffer was added for super-resolution imaging. dSTORM buffer was freshly prepared by combining glucose oxidase, catalyse and mercaptoethylamine (MEA; 50 mM PBS-Tris, 0.5 mM glucose, 1.3 μM glucose oxidase, 2.2 μμ catalase and 50 mM MEA). After diffraction-limited imaging, the slide was removed from the microscope and two additional gaskets (GRACE Bio-Labs CultureWell 50—3 mm diameter × 1 mm depth, Cat. 103350) were stacked on top of the slide to increase the well capacity before STORM buffer was added to each well. The slide was then sealed by applying a smaller coverslip over the top of the gaskets and applying nail varnish to the sides to produce an airtight seal. The slide was then returned to the microscope for STORM imaging.

### Microscope set-up

Image acquisition was completed using an in-house Total Internal Reflection Fluorescence (TIRF) microscope. A 100× 1.49 NA oil-immersion objective (UPLSAPO, 100×, TIRF, Olympus) was mounted on a Nikon Ti2 Eclipse inverted microscope body with a perfect focus system. An excitation laser beam (Oxxius, 638 nm) was circularly polarized by a quarter-wave plate (WPQ05M-405, Thorlabs) and focused onto the back focal plane of the objective. The fluorescent signal from samples was collected via the same objective separated by a dichroic beam splitter (Di01-R405/488/561/635, Semrock). The emission was filtered by a long-pass emitter (BLP01-635R-25, Laser 2000). An air-cooled EMCCD camera (Photometrics Evolve, EVO-512-M-FW-16-AC-110) with frame transfer mode (electron-multiplying gain of 11.5 e-1/ADU and 250 ADU/photon) was used for image recording. The open-source software Micro-Manager 1.4 was used to operate the imaging system and automate data acquisition. For diffraction-limited imaging, a 1.5 mW laser was used with a 50 ms exposure time to capture 50 frames per field of view (FoV). For dSTORM imaging, a 150 mW laser was applied and images were acquired using an exposure time of 15 ms to capture 8000 frames. The camera was operated in pre-exposure non-overlapping mode. Continuous illumination with a 405 nm laser (LBX-405-50-CIR-PP, Oxxius) was applied at 10 mW power. The pixel size of the image was 103.5 nm^2^. Each FoV contains an area of ∼2500 µm^2^. For each patient, 16 diffraction-limited and 4 dSTORM images were acquired.

### Image processing and statistical analysis

Diffraction-limited single-molecule counting was performed using a single-molecule localization engine in ThunderSTORM.^40^ The images were processed using a wavelet filter, and the localizations were identified using a hybrid threshold of 0.5 × std(Wave.F1) + 0.1 × mean(Med. F). This arrangement gives a higher resistance to noise by combing the adaptive threshold and the mean-value threshold.

dSTORM data were analysed using published ImageJ^41^ plug-ins, with an automation code integrating these independent libraries. The drift correction, image reconstruction and morphology analysis were performed by a mean shift algorithm,^42^ ThunderSTORM^40^ and morphology library,^43^ respectively. To avoid blurred images, which may be collected during the microscope focusing stabilization, the first 200 frames were removed before further analysis. The localizations were identified using ThunderSTORM, and drift correction was performed by the mean shift algorithm. The drift-corrected localizations were then filtered using localization merge, and a density filter was applied to remove false-positive signals. These processed localizations were then used to reconstruct the super-resolved image. Morphology analysis was then performed providing area and circularity measurements.

All FoVs were analysed for each participant. Prior to comparisons, data were checked for normality using Shapiro–Wilks test, and normally distributed data were tested for homogeneity of variance using Levene’s test. All data were compared using two-tailed tests. Where the variance between the two groups was significant, Welch’s *t*-test was applied; otherwise, Student’s *t*-test was used. For significant results that were parametric, the effect size was measured using Cohen’s *d*. Correlations were tested using Pearson rank test. Non-normally distributed data were compared using the Wilcoxon rank sum test, or for pairwise comparisons, Dunn’s test was applied. A correction for multiple analyses was made using Bonferroni’s method. For significant non-parametric results, the effect size (*r*) was calculated using $r=\;Z/\sqrt{N}$. Non-normal correlations were tested using Kendall tau test. Categorical variables were compared using Pearson’s *χ*^2^ test. The optimum cut-off values were determined using the value that provided the maximum sum of sensitivity and specificity. For logistic regression, *P*-values were calculated using Weld’s method, the model’s explanatory power was measured using McFadden’s pseudo *R*^2^ with significance assessed using *χ*^2^ test.

## Results

### Participants

We recruited 2 subgroups, each including 10 Parkinson’s disease and 10 control participants. The sample size per subgroup was limited by the available space on the imaging slide (40-well coverslip). Subgroups were grouped by the period of sample collection. Each subgroup was recruited over a different 3-month period and was analysed soon after the last sample was collected. Due to concerns regarding the effect of the duration of sample storage on aggregate number and structure and due to variation in absolute number of detected aggregates between experimental runs, we initially analysed each subgroup independently before later combining the subgroups and comparing ratio results. Demographic data for each subgroup are shown in Table 1. In Subgroup 1, participants with Parkinson’s disease were younger than controls (Parkinson’s disease = 67.04 years, control = 74.15 years), but otherwise, the Parkinson’s disease cases and controls were well matched across subgroups. Both subgroups included participants with early stage Parkinson’s disease as indicated by disease duration and Hoehn and Yahr score (</−3), but cases in Subgroup 1 had a slightly shorter disease duration than Subgroup 2 (1.09 versus 2.75 years). In keeping with previously reported longitudinal data,^34^ 20 and 40% of patients with Parkinson’s disease had MoCA scores suggestive of mild cognitive impairment (MCI; score <26) in Subgroups 1 and 2, respectively;^44^ no patients scored in the dementia ranges (score <21).

### Subgroup 1

#### Subgroup 1: number of aggregates

SiMPull was used to quantify the number of α-synuclein and Aβ aggregates present in saliva. First, we evaluated the sensitivity and specificity of the SiMPull assay for each target (Supplementary Figs. 1 and 2, Supplementary Tables 1 and 2). Three control conditions were used to ensure that capture was specific to the target protein aggregate, accounting for non-specific protein binding to the coverslip surface (capture control), non-specific detection antibody binding to the coverslip (blank control) and detection/capture antibody interactions (detection control). There was a clear signal distinction between the full antibody condition and the control conditions, indicating that the assay was specific for the detection of α-synuclein- and Aβ-containing aggregates. We propose that by using the same epitope-specific antibody for detection and capture, all localization points are dimers or larger given each monomer only contains a single-antibody-binding epitope.^45^

**Figure 2:**
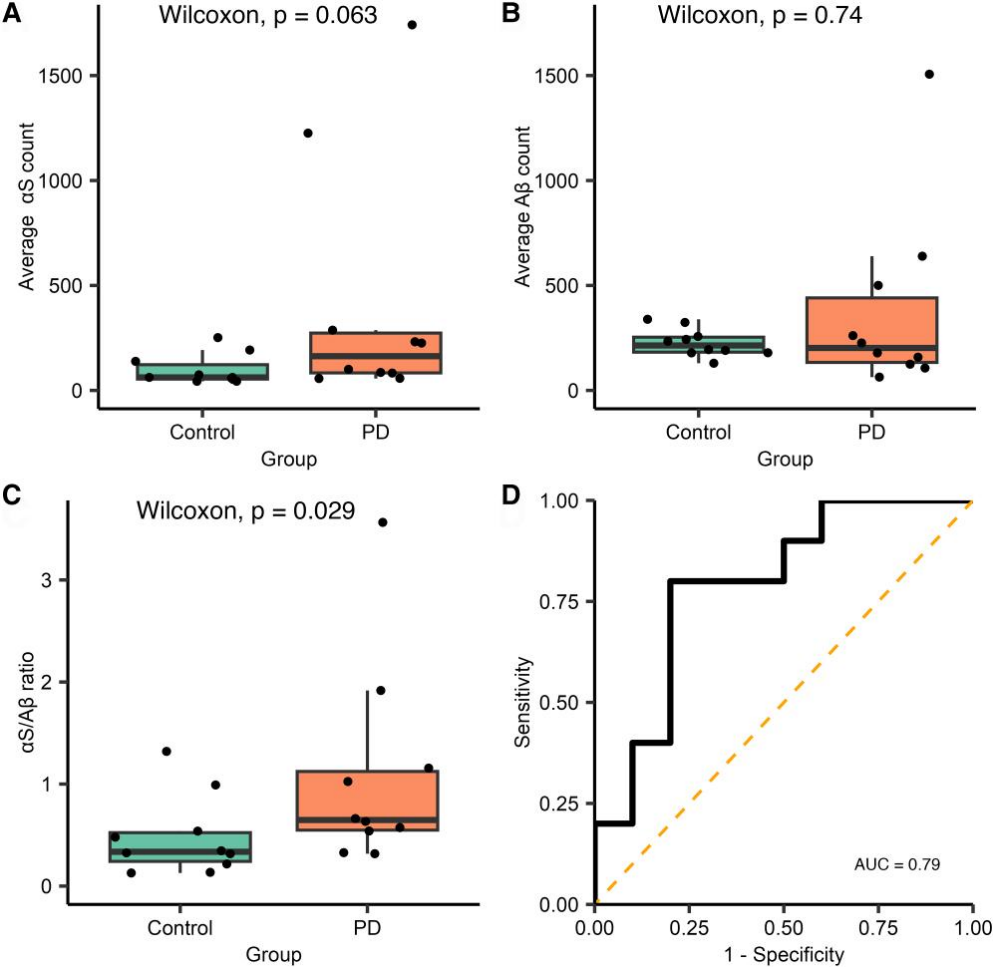
**Analysis of diffraction-limited single-aggregate counting for Subgroup 1.** Each FoV is 2500 µm^2^ and 16 fields of view were measured for each participant, Parkinson’s disease *n* = 10 and control *n* = 10. (**A**) A non-significant increase in the number of α-synuclein-containing aggregates was observed in the saliva of people with Parkinson’s disease compared with controls (*W* = 75, *P* = 0.063), and there was no difference in the number of Aβ-containing aggregates (*W* = 45, *P* = 0.74) (**B**). (**C**) The ratio of the number of α-synuclein to Aβ aggregates is significantly increased 2.2-fold in people with Parkinson’s disease compared with controls (*W* = 79, *P* = 0.029, *r* = 0.49). (**D**) ROC analysis of ratio values (AUC = 0.79). αS, α-synuclein; Aβ, amyloid-β; AUC, area under the curve; PD, Parkinson’s disease.

The antibody SiMPull assay was then used to compare the total aggregate count (Fig. 2, Supplementary Tables 3 and 4) in Parkinson’s disease (*n =* 10) and control groups (*n =* 10). There was a non-significant trend for increased α-synuclein, containing aggregates in the saliva of patients with Parkinson’s disease when compared with controls (median Parkinson’s disease *=* 164.03, control *=* 62.09, *W =* 75, *P =* 0.063), while there was no clear difference in the number of Aβ-containing aggregates (median Parkinson’s disease = 202.25, control *=* 214.14, *W =* 45, *P =* 0.74). Taking the ratio of α-synuclein to Aβ for each participant helps to reduce the effect of differences in total aggregate number in saliva between participants. The α-synuclein/Aβ ratio provides better discrimination between Parkinson’s disease and control groups with approximately a 2.2-fold increase in the saliva of participants with Parkinson’s disease [median Parkinson’s disease *=* 1.07, control *=* 0.48, *W =* 79, *P =* 0.029, 95% confidence interval (CI; 0.002–0.890), *r =* 0.49; Fig. 2C]. Receiver operator characteristics (ROCs) were used to evaluate the sensitivity and specificity of the α-synuclein/Aβ aggregate ratio for discriminating between the groups and yielded an area under the curve (AUC) of 0.79.

#### Subgroup 1: aggregate size and morphology

dSTORM was used to obtain super-resolved images of individual protein aggregates with a spatial resolution of ∼20 nm. Using this method, we can provide quantitative measurements of aggregate area and circularity (Parkinson’s disease *n =* 9, control *n =* 8). We have previously shown that our sample preparation allows the dSTORM assay and aggregates to remain stable over 12 h providing time for image acquisition overnight (Y. P. Zhang *et al.*, submitted for publication). Participants were excluded where the number of localizations were insufficient for drift correction. To analyse the morphology of salivary aggregates, we visualized the area and circularity distributions for each aggregate type according to disease status using their cumulative distributions. Across both groups, aggregate area measurements had a large positive skew, while circularity measurements were normally distributed (Supplementary Table 5). The median area for α-synuclein-containing aggregates from Parkinson’s disease and control saliva was 0.005 µm^2^ with an interquartile range (IQR) of 0.011 and 0.010 µm^2^, respectively; the mean circularity was 0.572 [standard deviation (SD) = 0.216] and 0.0578 (SD = 0.217), respectively. Aβ-containing aggregates from Parkinson’s disease and control saliva had a median area of 0.006 µm^2^ with an IQR of 0.013 and 0.011 µm^2^, respectively; the mean circularity was 0.576 (SD = 0.211) and 0.614 (SD = 0.192). The comparison of mean values gives an indication of the impact of the positive skew on the overall distribution. There was no difference in the mean α-synuclein-containing aggregate area between groups (0.012 µm^2^). In contrast, the mean area of Aβ-containing aggregates in Parkinson’s disease saliva (0.013 µm^2^) was higher than in controls (0.011 µm^2^). This suggests that the overall distribution of aggregate area may differ between the groups with a more significant subpopulation of larger Aβ aggregates in the Parkinson’s disease group resulting in a more positively skewed mean area.

**Figure 3:**
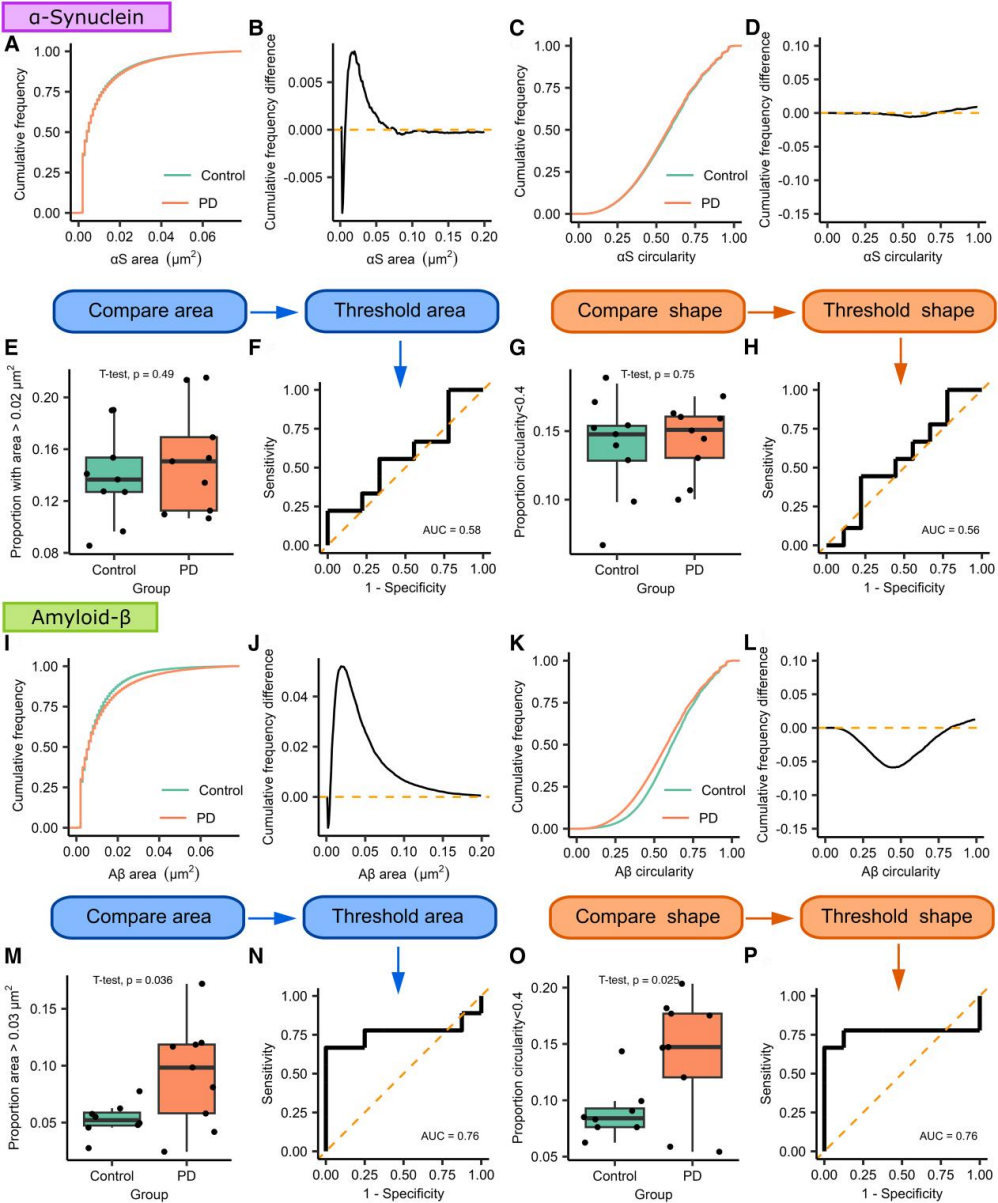
**Morphological analysis of α-synuclein and Aβ-containing aggregates from Subgroup 1 using dSTORM super-resolution imaging.** For both types of aggregates, we compare the cumulative frequency curves for the morphological feature of interest. The two distributions are subtracted from each other to demonstrate how the two curves differ; the point of maximum difference is then used as a threshold to distinguish groups of morphologically distinct aggregates. (**A–H**) α-Synuclein. We find no difference in the size (area, **A, B, E**) or shape (circularity, **C, D, G**) of α-synuclein-containing aggregates (Parkinson’s disease *n* = 9, control *n* = 9). For Aβ-containing aggregates (**I–P**, Parkinson’s disease *n* = 9, control *n* = 8), we show that the area distribution differs between the two groups and that the two groups maximally differ from each other at 0.03 µm^2^ (**I, J, M**). Using this value as a cut-off, we show that saliva from patient with Parkinson’s disease contains a greater proportion of aggregates >0.03 µm^2^ (*t*(9.75) = 2.43, *P* = 0.036, *d* = 1.15). ROC analysis demonstrates that aggregate size can distinguish between Parkinson’s disease and controls (**N**, AUC = 0.76). Shape data analysis shows a visible difference between circularity distributions (**K**, **L**, **O**), we find that the two distributions are maximally different from each other at a circularity value of 0.4 (*t*(15) = 2.48, *P* = 0.025, *d* = 1.23), and ROC analysis shows that shape data can distinguish Parkinson’s disease from controls (**P**, AUC = 0.76). αS, α-synuclein; Aβ, amyloid-β; AUC, area under the curve; PD, Parkinson’s disease.

To determine whether morphology information could distinguish between patients with Parkinson’s disease and controls, we utilized a similar workflow to that described by Zhang *et al.*^31^ to isolate subpopulations of aggregates that differed between groups based on area and circularity (Fig. 3). We subtracted the Parkinson’s disease cumulative frequency distribution from the control group distribution to identify the point at which the morphological feature was maximally different. Once identified, we then used this value as threshold from which we identified the proportion of aggregates that were larger, or less circular than the identified cut-off for each participant. The proportion values were then compared to see whether there was a significant difference between the two groups (Supplementary Tables 3 and 4).

When comparing the area for the α-synuclein-containing aggregates, we found a small divergence between the two distributions at 0.02 µm^2^. However, when using this value as a threshold, we found no difference in the proportion of α-synuclein-containing aggregates >0.02 µm^2^ between the two groups (*t*(16) = −0.709, *P* = 0.489). Similarly, when comparing circularity, a small difference between distributions was found for aggregates less circular than 0.4 with no significant difference between the two groups in the proportion of aggregates satisfying this threshold (*t*(16) = −0.329, *P* = 0.747).

For Aβ-containing aggregates, we found a maximal difference between the two area distributions at 0.03 µm^2^. Using this as a threshold, we showed a significantly greater proportion of Aβ-containing aggregates >0.03 µm^2^ in the Parkinson’s disease group (*t*(9.75) = 2.43, *P* = 0.036, *d =* 1.15), applying this area threshold can be used to distinguish between the two groups with an AUC of 0.76. Similarly, for circularity, the point of maximum difference was 0.4. There was a significantly greater proportion of Aβ aggregates less circular than 0.4 in the Parkinson’s disease group (*t*(15) = 2.48, *P* = 0.025, *d* = 1.23) and this threshold distinguishes between the two groups with an AUC of 0.76. This, therefore, suggests that Aβ-containing aggregates in the Parkinson’s disease group are larger and more fibrillar than those observed in the control group and that morphology data can be used to distinguish between the two groups with a reasonable degree of accuracy.

#### Subgroup 1: combining aggregate count and size data

**Figure 4:**
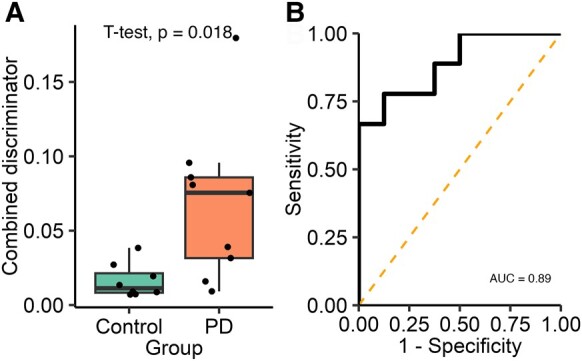
**Analysis of the combined single-aggregate count and super-resolution morphological data for Subgroup 1.** A combined discriminator is calculated for each participant by multiplying the α-synuclein/Aβ aggregate ratio by the proportion of Aβ-containing aggregates satisfying both morphological feature thresholds (area >0.03 µm^2^ and circularity <0.4, Parkinson’s disease *n* = 9, control *n* = 8). (**A**) The combined discriminator is significantly higher in the PD group (4.3-fold increase, *t*(8.83) = 2.88, *P* = 0.018, *d* = 1.36), (**B**) applying ROC analysis demonstrates that this metric can accurately distinguish between Parkinson’s disease and control participants (AUC = 0.89). AUC, area under the curve; PD, Parkinson’s disease.

To determine whether combining diffraction-limited count with super-resolution morphology data might provide a greater discrimination between the two groups, we developed a combined discriminator. First, for each participant, we found the proportion of aggregates that satisfied both morphology thresholds previously identified (area >0.03 µm^2^ and circularity <0.4) and then multiplied this proportion to the α-synuclein/Aβ ratio found using diffraction-limited imaging. Using this combined discriminator (Fig. 4), we show a significant difference between the two groups [*t*(8.83) = 2.88, *P* = 0.018, 95% CI (0.013–0.095), *d* = 1.36] with a 4.3-fold increase in the Parkinson’s disease saliva. The combined discriminator also provides a greater ability to distinguish between Parkinson’s disease and control groups than either count or morphology data alone providing an AUC of 0.89. The optimum cut-off value identified was 0.039 which provides a sensitivity of 66.6% and a specificity of 100%.

### Subgroup 2

#### Subgroup 2: number of aggregates

**Figure 5:**
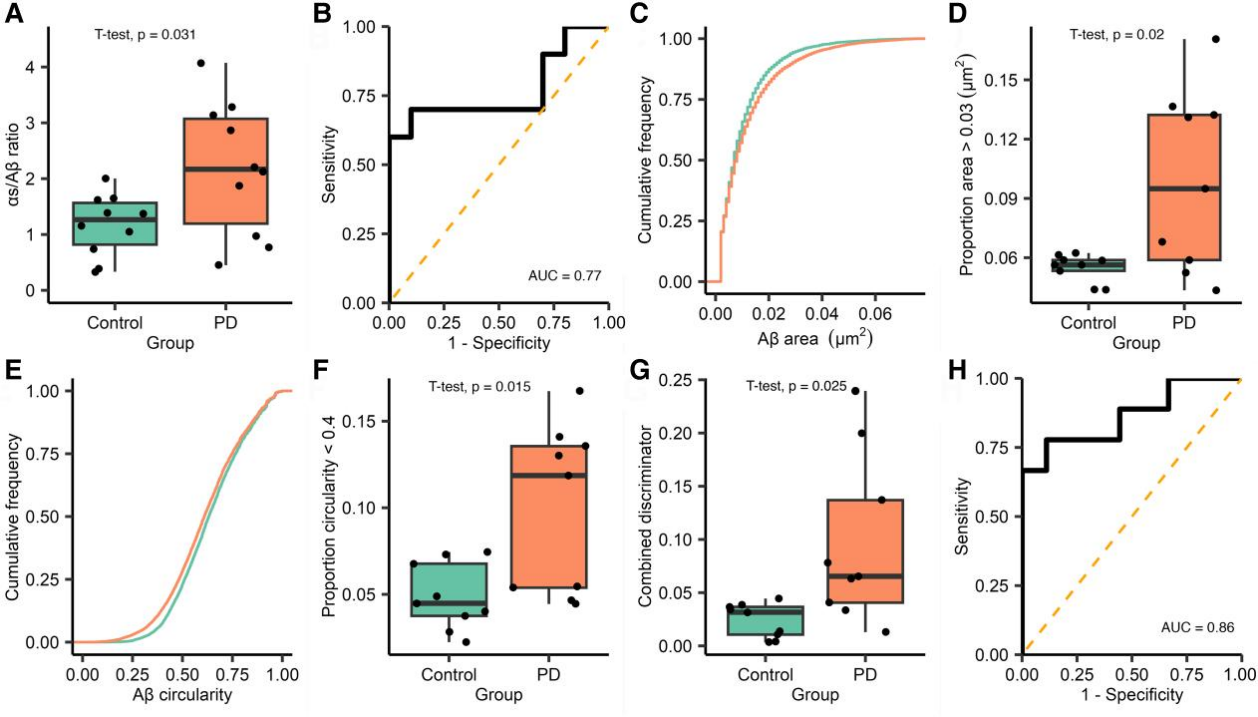
**Analysis of diffraction-limited single-molecule aggregate counting, super-resolution morphological data and combined discriminator data for Subgroup 2.** For diffraction-limited data, each FoV is 2500 µm^2^ and 16 fields of view a captured for each participant, *n* = 10 Parkinson’s disease and 10 control. (**A**) The ratio of the number of α-synuclein to Aβ aggregates is significantly higher in patients with Parkinson’s disease (1.9-fold increase, *t*(12.68) = 2.42, *P* = 0.031, *d* = 1.08). (**B**) ROC analysis of diffraction-limited ratio values (AUC = 0.77). For super-resolution data, analysis was completed as described in Fig. 3 (Subgroup 2 Parkinson’s disease *n* = 9 and control *n* = 9). (**C**) For Aβ-containing aggregates, we show that there is a different area distribution between the two groups, applying the threshold values identified in Subgroup 1 and (**D**) we show that there is significantly more Aβ aggregates >0.03 µm^2^ in the Parkinson’s disease group (*t*(8.365) = 2.852, *P* = 0.02, *d* = 1.345). (**E**) For Aβ shape data, analysis shows a visible difference between circularity distributions, the Parkinsons disease group has more Aβ with a circularity <0.4 (**F**, *t*(10.43) = 2.901, *P* = 0.015, *d* = 1.367). Full analysis, including α-synuclein data, is shown in Supplementary Fig. 4. A combined discriminator was calculated as previously described in Fig. 4. (**G**) The combined discriminator is significantly higher in the Parkinson’s disease group (4-fold increase, *t*(10.43) = 2.901, *P* = 0.025, *d* = 1.367), (**H**) applying ROC analysis demonstrate that this metric can accurately distinguish between Parkinson’s disease and controls (AUC = 0.86). Aβ, amyloid-β; AUC, area under the curve; PD, Parkinson’s disease.

Similarly to Subgroup 1, we found a non-significant trend for an increased number of α-synuclein-containing aggregates within the saliva from patients with Parkinson’s disease (mean Parkinson’s disease = 166.25, control = 124.23, *t*(18) = 1.44, *P* = 0.167; Supplementary Fig. 3A) and no significant difference in the number of Aβ-containing aggregates (median Parkinson’s disease = 84.50, control = 111.22, *W* = 39, *P* = 0.436, Supplementary Fig. 3B). Taking the α-synuclein/Aβ aggregate count ratio showed a 1.9-fold increase in the relative number of α-synuclein-containing aggregates in the Parkinson’s disease group [mean Parkinson’s disease = 2.18, control = 1.17, *t*(12.68) = 2.42, *P* = 0.031, 95% CI (0.107–1.902), *d* = 1.08, Fig. 5A], although this difference was less marked than that seen in Subgroup 1. Applying ROC gave an AUC of 0.77 (Fig. 5B).

#### Subgroup 2: aggregate size and morphology

We applied dSTORM imaging to the captured aggregates from the second subgroup. Aggregate morphology data are summarized in Supplementary Table 5 (Parkinson’s disease *n* = 9, control *n* = 9). Participants were excluded where the number of localizations were insufficient for drift correction. The pooled data across Parkinson’s disease and controls for each aggregate were marginally larger and rounder than those observed in Subgroup 1. Again, a positive skew was seen in the distributions of area values for both aggregate types, while circularity data were normally distributed. The mean area of Aβ-containing aggregates was higher in the Parkinson’s disease group.

To determine whether the threshold values identified in the first subgroup generalized across participants, we applied the same threshold values for area (α-synuclein >0.02 µm^2^, Aβ > 0.03 µm^2^) and circularity (<0.4) to the second subgroup (Fig. 5C–F, Supplementary Fig. 4). Using these thresholds, we found no significant difference in the proportion of α-synuclein-containing aggregates >0.02 µm^2^ (*t*(15) = 0.901, *P* = 0.382) and no difference in the proportion less circular than 0.4 (*t*(15) = 1.157, *P* = 0.265). In contrast, for Aβ-containing aggregates, we again showed a significantly greater proportion of aggregates >0.03 µm^2^ (*t*(8.365) = 2.852, *P* = 0.02, *d* = 1.345), and when used to separate the two groups, this gave an AUC of 0.77. Additionally, there was a significantly greater proportion of Aβ-containing aggregates with a circularity <0.4 in the Parkinson’s disease group (*t*(10.43) = 2.901, *P* = 0.0152, *d* = 1.367) providing an AUC of 0.81.

#### Subgroup 2: combining aggregate count and size data

Finally, we combined the combined count and morphology data to produce a combined discriminator. As in Subgroup 1 using this value gave a clear difference between the two groups [4-fold increase in Parkinson’s disease, *t*(8.669) = 2.713, *P* = 0.0247, 95% CI (0.012–0.133), *d* = 1.279; Fig. 5G and H] and allowed them to be accurately distinguished from each other, AUC = 0.86. The optimum cut-off value was identified as 0.0517 which produced a sensitivity of 66.6% and a specificity of 100%.

### Combined subgroups

#### Discrimination between Parkinson’s disease and controls

**Figure 6:**
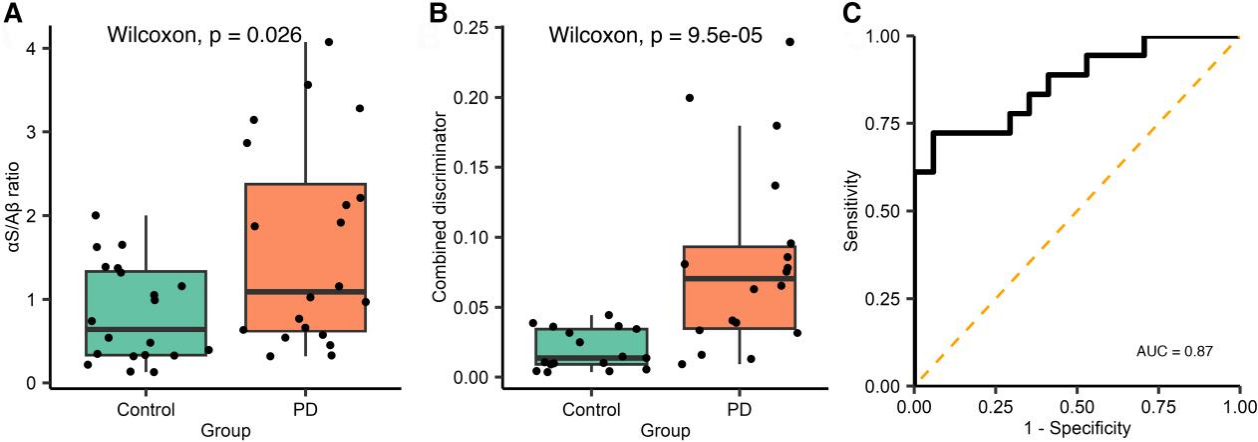
**Analysis of combined single-molecule aggregate count data and super-resolution morphological data for data combined across Subgroups 1 and 2.** (**A**) The α-synuclein/Aβ aggregate ratio was significantly higher in when the subgroups were combined. (**B**) The combined discriminator was calculated, as previously described (Parkinson’s disease *n* = 18, control *n* = 17), the combined discriminator was increased 5-fold in the Parkinson’s disease group (*W* = 41, *P* < 0.001). (**C**) Applying ROC analysis demonstrated that this metric can accurately distinguish between Parkinson’s disease and controls (AUC = 0.86). αS, α-synuclein; Aβ, amyloid-β; AUC, area under the curve; PD, Parkinson’s disease.

Currently, the sample size of each subgroup is limited by the number of wells available on the imaging slide. We have found that total aggregate counts vary between individual experimental runs, whereas ratio values are more comparable. To maximize our sample size, we combined the two subgroups (Supplementary Table 7). Diffraction-limited α-synuclein/Aβ aggregate ratio (Parkinson’s disease *n* = 20, control *n* = 20) was significantly higher in the Parkinson’s disease group [*W* = 118, *P* = 0.026, 95% CI (0.094–1.492), *r* = 0.351]. There was a 5-fold increase in the combined discriminator for Parkinson’s disease saliva compared with controls, the combined discriminator had a high degree of accuracy in distinguishing between people with Parkinson’s disease (*n* = 17) and controls [*n* = 18, *W* = 41, *P* < 0.001, 95% CI (0.0244–0.0727), *r* = 0.63, Fig. 6], AUC = 0.87. Using a 0.039 combined discriminator cut-off gave a sensitivity of 72.2% and a specificity of 94.1%.

In both Subgroup 1 and the combined data, the control group was significantly older than the Parkinson’s disease group (Supplementary Table 8). To assess the impact of age on the predictive value of the combined discriminator, we constructed two models using logistic regression and analysed the combined subgroup data (Supplementary Table 9). The first used only the combined discriminator to predict disease state, and the second combined age and the combined discriminator. We found that age had a non-significant effect on the model’s explanatory power. This was also reflected in the measures of model fit; the combined discriminator alone performed well (pseudo *R*^2^  *=* 0.376, *P =* 0.2 × 10^−4^) and including age provided minimal improvement (pseudo *R*^2^ = 0.396, *P* = 0.1×10^−4^) suggesting that the combined discriminator remains predictive of disease state when age is considered.

#### Correlation between aggregate measures and clinical features

Using the combined data, we investigated whether there was any correlation between the α-synuclein/Aβ ratio (*n* = 40), Aβ area and circularity thresholded proportions (*n* = 35) and the combined discriminator with demographic data and clinical scores (Table 2).

**Table 2:** Tests for correlation between patient demographic and clinical scores and measures of α-synuclein/Aβ ratio, proportion of Aβ aggregates meeting morphology thresholds (threshold Aβ) and the combined discriminator

Combined PD and control subgroup data									
	Ratio			Threshold Aβ			Combined discriminator		
	Cor	Stat (Kendall)	*P*-value	Cor	Stat (Kendall)	*P*-value	Cor	Stat (Kendall)	*P*-value
Age (years)	−0.250	291	0.021	−0.059	280	0.632	−0.320	202	0.006
*Controls only*									
Age (years)	−0.032	92	0.873	0.085	83	0.654	−0.190	55	0.308
*PD only*									
Age (years)	−0.260	70	0.113	0.004	0.017	0.987	−0.2	61	0.260
MDS-UPDRS-III	−0.048	−0.292	0.770	0.018	0.074	0.942	0.026	0.152	0.879
MDS-UPDRS Total	−0.005	−0.0325	0.974	0.16	0.656	0.521	0.15	0.782	0.383
Total ACE-III	0.24	1.454	0.146	−0.085	−0.340	0.736	0.18	0.999	0.318
Total MoCA	0.12	0.692	0.489	0.21	0.862	0.402	0.17	0.961	0.336

We found a statistically significant but weak negative correlation between age and the α-synuclein/Aβ ratio (*r_τ_* = −0.250, *P* = 0.021) and the combined discriminator (*r_τ_* = −0.320, *P* = 0.006). Within the Parkinson’s disease group, there was no correlation between any of our measures and MDS-UPDRS-III, MDS-UDPDRS-Total or MoCA scores, which is unsurprising given the small sample size.

#### Protein concentrations

To determine whether our measurements relate to more established neurodegeneration-related proteins, we used commercial ELISA kits to measure the total α-synuclein, Αβ_40_ and Αβ_42_ protein concentrations for participants where there was a sufficient sample (Supplementary Table 6). As shown in Supplementary Fig. 5, there was no difference in the concentration of α-synuclein (Parkinson’s disease *n* = 15, healthy control (HC) *n* = 16, *t*(24.174) = 0.446, *P* = 0.660), Aβ_40_ (Parkinson’s disease *n* = 16, HC *n* = 17, *W* = 103, *P* = 0.242), Aβ_42_ (HC *n* = 19, Parkinson’s disease *n* = 19, *W* = 201.5, *P* = 0.549) or Aβ_42_/Aβ_40_ ratio (Parkinson’s disease *n* = 16, HC *n* = 17, *W* = 171, *P* = 0.217) between Parkinson’s disease and control groups. We also determined whether aggregate count taken as a proportion of total protein concentration varied between Parkinson’s disease and controls. We showed no difference in α-synuclein count per ng/ml total α-synuclein (*W* = 78, *P* = 0.101), Aβ_40_ count per pg/ml (*W* = 151, *P* = 0.606) or Aβ_42_ count per pg/ml (*W* = 190, *P* = 0.795).

## Discussion

Our results show that we can sensitively and specifically detect disease relevant protein aggregates in saliva at a single-molecule level. Using an aggregate ratio value allows us to distinguish patients with Parkinson’s disease from controls with a high degree of accuracy. By using the same antibody for detection and capture, we capture aggregated protein forms; this is also reflected in the size data from our super-resolution experiments. As there was no evidence of a difference in the total number of Aβ-containing aggregates between the groups, this suggests that there is a greater relative abundance of α-synuclein-containing aggregates in saliva from patients with Parkinson’s disease compared with controls. Using ELISA, we show that there is no difference in the total concentration of α-synuclein, this suggests that the increased aggregate count is due to a shift from monomeric to aggregated forms. Our finding of increased relative levels of α-synuclein aggregates is, therefore, in agreement with previous studies finding elevated levels of oligomeric α-synuclein in the saliva of patients with Parkinson’s disease.^19,20,25^ In the pooled data, age is significantly lower in the Parkinson’s disease group, and age also has a weak negative correlation with the combined discriminator raising the possibility that the observed difference in the combined discriminator may be confounded by age. Using a regression model, we have, however, shown that age does not have a statistically significant effect on its predictive performance, thus the combined discriminator is the key variable that predicts group and hence Parkinson’s disease status.

The majority of previous studies that utilized ELISA-based methods to measure salivary α-synuclein did not report ROC analysis making comparison difficult.^18,19,21,25^ One study that used ELISA to measure total and oligomeric α-synuclein reported that the ratio value provided a sensitivity of 69.77% and specificity of 95.16% but does not report the method used to determine the optimum cut-off threshold.^20^ Our SiMPull method provides comparable results with both Subgroups 1 and 2 reporting a sensitivity 66.6% and a specificity of 100% when using a sum of sensitivity and specificity to identify the optimum cut-off value. The sensitivity and specificity from the pooled data across both subgroups are 72.2 and 94.1%, respectively. In our study, we do not find any difference in total α-synuclein using ELISA, this suggests that single-molecule characteristics, detected using SiMPull, provide greater sensitivity than total protein concentration measures. More recently, SAA has been used to compare the seeding activity of α-synuclein present in saliva, and these studies have reported an AUC of 0.84–0.9.^27,28^ For both subgroups, our SiMPull method performs towards the upper end of these values (Subgroup 1 AUC = 0.89, Subgroup 2 AUC = 0.86) while having the advantage of allowing easy target customization as illustrated by quantifying both α-synuclein and Aβ within the same imaging slide.

Our imaging approach has the advantage of allowing the same aggregates captured and counted during diffraction-limited SiMPull experiments to be subsequently super resolved with minimal additional processing. This means that we can obtain both single-molecule abundance data and morphology data for the same captured aggregates within a single experimental condition. Even though saliva is a protease rich environment, we were able to find measurable differences in aggregate morphology between groups, but unexpectedly, this difference was observable in Aβ-containing aggregates as opposed to α-synuclein. We propose this may be due to the detection of Aβ co-pathology or due to differences in protease resistance between α-synuclein and Aβ.

Changes in Aβ aggregation have been shown to be relevant to Parkinson’s disease, particularly in relation to cognition. Longitudinal studies show MCI to be present at diagnosis in ∼20–30% of patients with Parkinson’s disease and progression to dementia in 50% after 10 years of follow-up.^34,46-50^ In keeping with previous studies, ∼30% of patients with Parkinson’s disease in our subgroups showed evidence of Parkinson’s disease MCI on MoCA testing. In Parkinson’s disease, CSF Aβ42 declines with worsening cognition,^22,33,34,51^ and these changes are predictive of future decline in cognitively normal individuals.^52^ No previous studies have described salivary Aβ within a population with Parkinson’s disease but studies examining the relationship between serum Aβ and cognition have shown variable results.^53-57^ Outside of cognition, it has also been suggested that Aβ may correlate with gait disturbance in Parkinson’s disease^35^ and that Aβ and α-synuclein act synergistically to enhance oligomer formation^58^ providing further evidence for the important role of Aβ co-pathology in Parkinson’s disease. In our study, we used 6E10 to measure Aβ. This antibody is relatively non-specific and binds to a range of Aβ isoforms including Aβ40, Aβ42 and APP fragments.^59^ This allows us to detect a broad range of aggregates facilitating their separation based on morphology but limits our ability to relate detected differences to specifically Aβ_42_ which has been implicated in disease. In addition, our ELISA studies are unable to detect differences in the concentration of Aβ_42_ limiting our ability to comment on the potential composition of the morphologically distinct Aβ subpopulation that we detect.

An alternative explanation for the lack of difference in α-synuclein morphology between groups could be provided by the biofluid studied and the antibodies used. Saliva is a complex biofluid that contains a range of different types of proteases including serine proteases^60,61^ which have been shown to have proteolytic effect on Aβ and α-synuclein. We used LB509 to detect α-synuclein which binds to Residues 115–122 of the C-terminus. This region is more vulnerable to protease cleavage^62^ which may reduce antibody binding, limiting our α-synuclein morphological analysis. This is in contrast to 6E10, which is relatively non-specific and, therefore, captures a greater variety of aggregates.^59^ Aβ aggregates faster than α-synuclein;^63^ however, the total Aβ concentrations in our samples are low, meaning that it is unlikely that aggregation occurring during sample preparation^64,65^ explains why we observe a difference in Aβ but not α-synuclein morphology.

The presented work has several limitations. Our low sample size is in part due to the throughput of our methodology, which is a limitation of the current study and the future use of our method more generally. To overcome this, we are developing a robotic system using a 96-well microplate that would allow for larger studies. In addition, detection intensities can be used in lieu of dSTORM imaging to indirectly provide a measure of aggregate size but with a far shorter imaging time.^31^ Our current super-resolution method is restricted to quantifying area and circularity and does not fully describe aggregate structure, which will limit our ability to detect differences between groups. Importantly, we detect differences in Aβ using the 6E10 antibody, which limits our ability to relate detected differences to disease relevant Aβ isoforms.

Although saliva offers several potential benefits, including ease of collection that could allow for self-sampling, the current sample processing protocol is labour intensive. Saliva collection can also be difficult to collect in the population with Parkinson’s disease, particularly for those with axial or jaw tremors, although this could feasibly be overcome with a better design of collection equipment. Future work should aim to assess which factors in collection (e.g. time of day) and processing substantially influence results.

## Conclusion

This study demonstrates that disease-relevant proteins present in saliva can be detected and characterized using single-molecule and super-resolution imaging techniques. Our results suggest that structural changes detected may be relevant to disease and may represent a potential new means for Parkinson’s disease diagnosis with performance that is superior to ELISA-based methods and comparable with SAA methods. Although our results are limited by our small sample size, future work utilizing larger automated microplates to facilitate greater sample sizes could be used to test this hypothesis.

## Supplementary Material

fcae178_Supplementary_Data

## Data Availability

Code used for image analysis is available at https://github.com/YPZ858/DF-single-molecule-couting- (single-molecule counting) and https://github.com/YPZ858/Super-res-code/issues (super-resolution imaging). Data obtained from image analysis and participant data are available at https://gin.g-node.org/MFurlepa/SalivaAggregates.git. For the purpose of open access, the authors have applied a Creative Commons Attribution (CC BY) licence to any author-accepted manuscript version arising from this submission.
