# Supplementary material for: Single-molecule characterization of salivary protein aggregates from Parkinson’s disease patients: a pilot study
Source: Brain Commun. 2024 May 21;6(3):fcae178. doi: 10.1093/braincomms/fcae178 (PMC11166177; doi:10.1093/braincomms/fcae178)
Supplement: fcae178_Supplementary_Data [file fcae178_supplementary_data.docx]

**Supplementary Information**

## **Supplementary Figures**


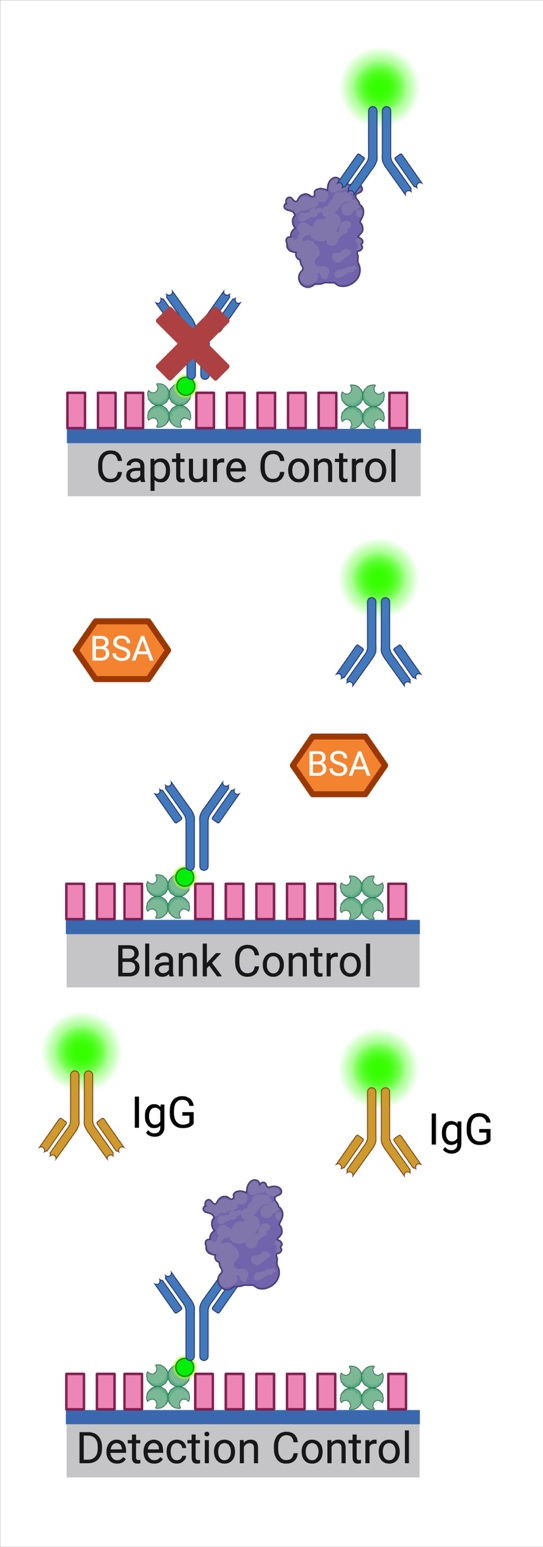


**Supplementary Figure 1 Illustration of control conditions examined for SiMPull validation experiments.**


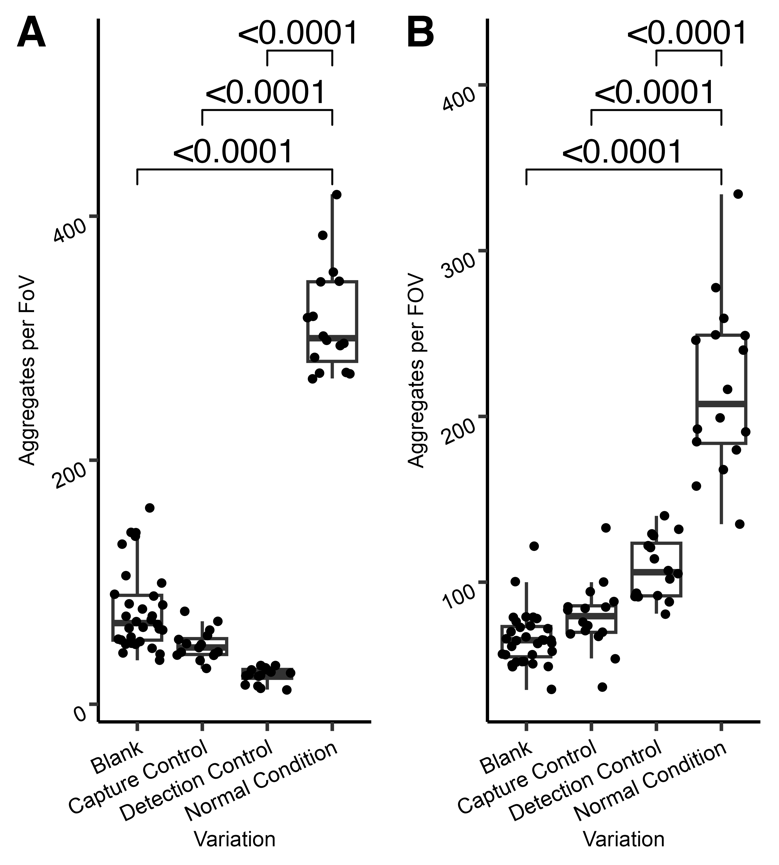


**Supplementary Figure 2** **Comparison of aggregate count across control conditions (Supplementary Fig. 1).** Saliva samples from a representative PD and control participant are included in the comparison. Box plots indicate the distribution of data for multiple FOV from the same well and across wells within the same imaging slide: capture control / detection control / normal condition n= 16, blank n = 32. Capture control omits capture antibodies and evaluates non-specific binding of aggregates and antibodies to the coverslip surface, detection control uses non-specific IgG antibodies for detection and controls for antibody-antibody interactions as well as non-specific detection antibody binding, blank control condition uses PBS in the place of saliva and acts as a negative control. Although most results were normally distributed, data from the blank group had a non-normal distribution. All data was therefore compared using more conservative non-parametric Dunn’s test with Bonferroni correction. A – Comparisons using LB509 antibody: the normal condition produced a higher signal than the capture control (Z = 5.136, p <0.001); detection control (Z = 7.761, p < 0.001); and blank control (Z = 1.449, p < 0.001). B – Comparisons using the 6E10 antibody: the normal condition produced a higher signal than the capture control (Z = 4.496, p <0.001); detection control (Z = 2.313, p < 0.001); and blank control (Z = 7.235, p < 0.001)


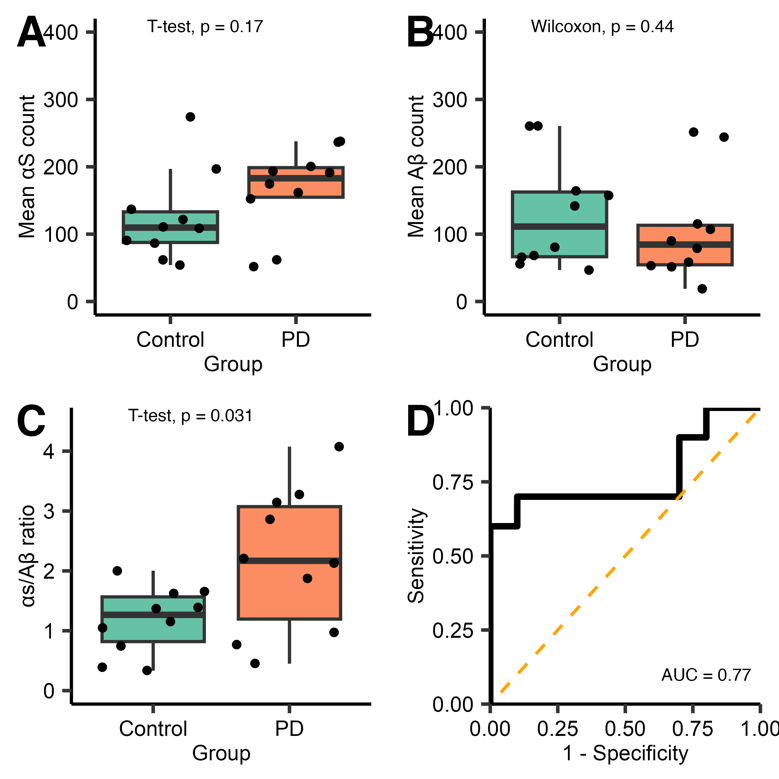


**Supplementary Figure 3** **Analysis of diffraction limited single molecule aggregate counting for subgroup two.** Each field of view is 2500µm^2^ and 16 fields of view a captured for each participant, n = 10 PD and 10 control. A - There is a non-significant increase in the number of α-synuclein containing aggregates present in the saliva of patients with PD (*p=0.17*) but no difference in the number of Aβ containing aggregates (B). C - the ratio of the number of α-synuclein to Aβ aggregates is significantly higher in patients with PD (1.9-fold increase, *p=0.031*). D – ROC analysis of ratio values (*AUC=0.77*)


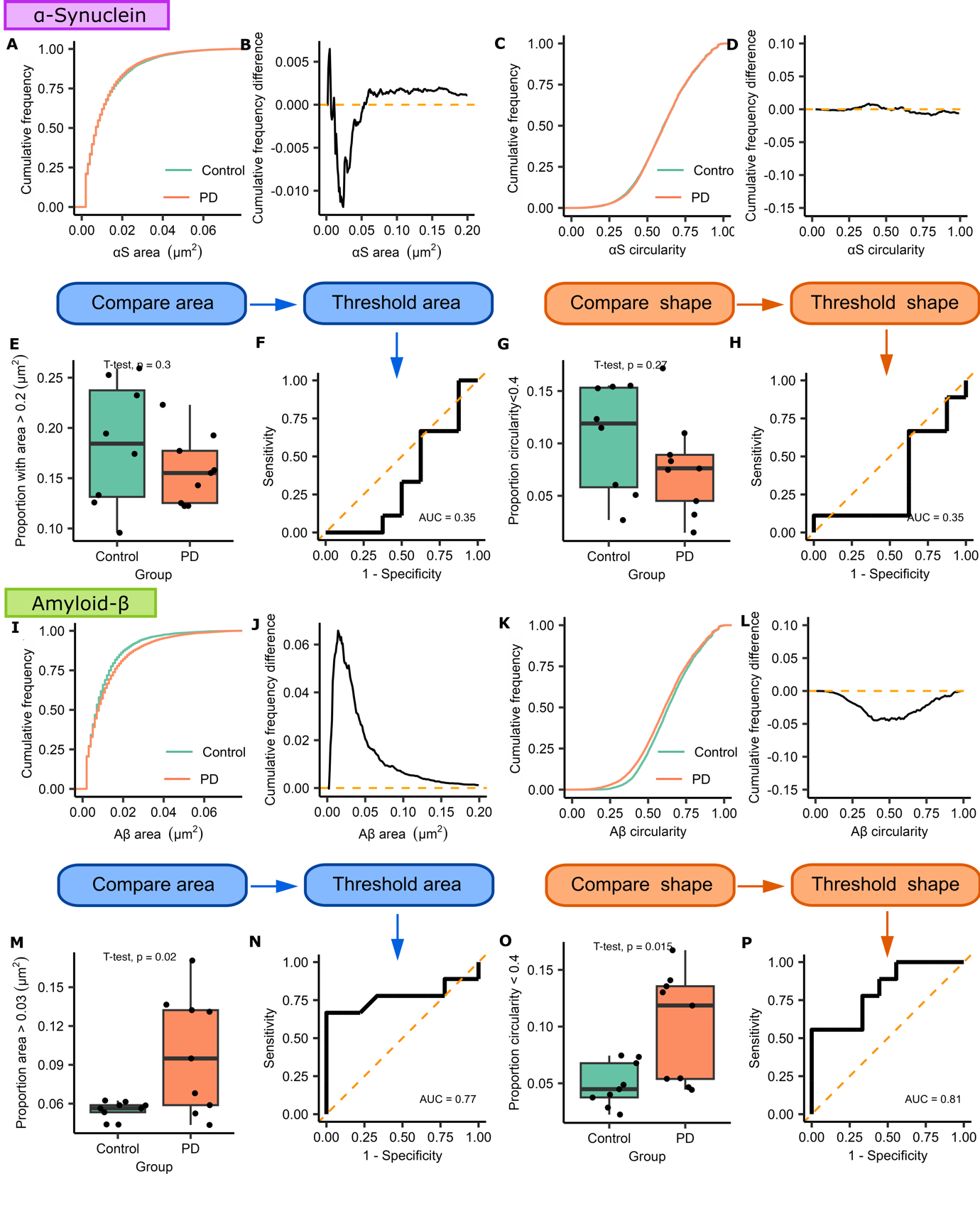


**Supplementary Figure 4** **Morphological analysis of α-synuclein and Aβ containing aggregates from subgroup two using dSTORM super-resolution imaging.** For both types of aggregate we compared the cumulative frequency curves for the morphological feature of interest. The two distributions are subtracted from each other to demonstrate how the two curves differ. We applied the morphology thresholds previously identified in subgroup 1 to subgroup 2 as described below. There is no difference in the proportion of α-synuclein aggregates with an area >0.02µm^2^ (A-B, E-F, *t(15) = 0.901, p = 0.382*) or with a circularity <0.4 (C-D, G-H, *t(15) = 1.157, p = 0.265*). For Aβ containing aggregates, we show that PD saliva contains a greater proportion of aggregates >0.03µm^2^ (I-J, M, *t(8.365) = 2.852, p = 0.02, d = 1.345*), ROC analysis demonstrates that aggregate size can distinguish between the PD and controls (N, *AUC=0.77*). Applying a circularity threshold of <0.4 shows a greater proportion of more fibrillar Aβ containing aggregates in people with PD (*K-L, O, t(10.43) = 2.901, p = 0.0152, d = 1.367*), and ROC analysis shows that shape data can distinguish PD from controls (P, *AUC=0.81*)


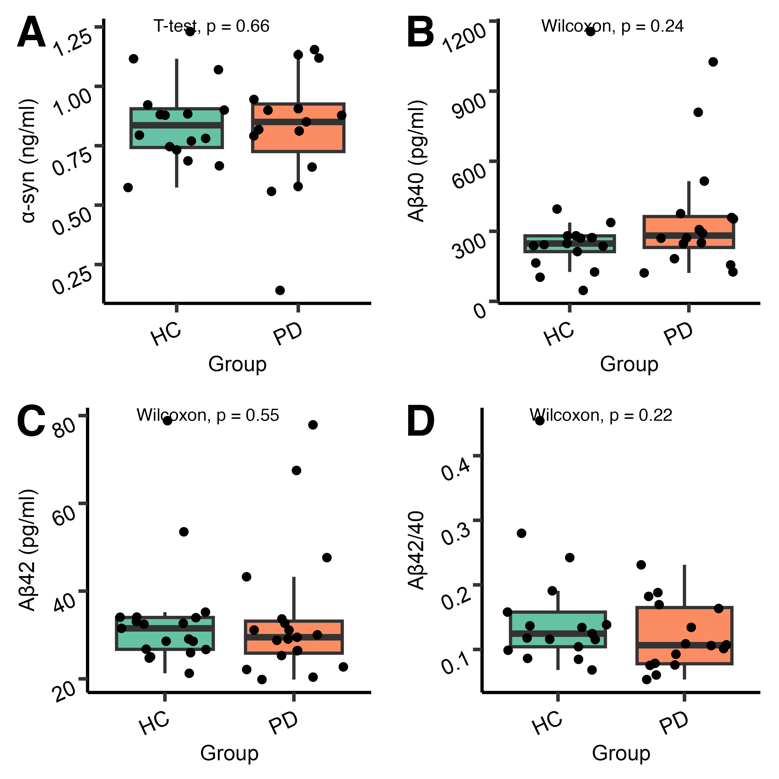


**Supplementary Figure 5** **Comparison of protein concentrations measured using ELISA.** there was no difference in the concentration of A - α-synuclein (PD n = 15, HC n = 16, t(24.174) = 0.446, p = 0.660), B - Aβ_40_ (PD n = 16, HC n = 17, W = 103, p = 0.242), C - Aβ_42_ (HC n = 19, PD n = 19, W = 201.5, p = 0.549), or D - Aβ_42_/Aβ_40_ ratio (PD n = 16, HC n = 17, W = 171, p = 0.217) between PD and HC groups

## **Supplementary Tables**

**Supplementary Table 1 normality tests (Shapiro-Wilks) for statistical comparisons of validation data (Supplementary Fig. 2, Supplementary Table 2)**

| **Normality tests** | **Full Condition** | **Blank Control** | **Detection Control** | **Capture Control** |
| --- | --- | --- | --- | --- |
| LB509 | 0.145 | <0.001 | 0.0709 | 0.797 |
| 6E10 | 0.729 | <0.001 | 0.324 | 0.294 |

**Supplementary Table 2 pairwise comparisons of saliva validation experiments, control conditions are compared to the full antibody condition (normal condition) see Supplementary Fig. 1-2.**

| **Pairwise comparisons** | **LB509 PD** | | **6E10 PD** | |
| --- | --- | --- | --- | --- |
|  | **p** | **Z** | **p** | **Z** |
| Capture control | <0.001 | 5.136 | <0.001 | 4.496 |
| Detection control | <0.001 | 7.761 | <0.001 | 2.313 |
| Blank | <0.001 | 1.449 | <0.001 | 7.235 |

**Supplementary Table 3 Summary statistics and group comparisons of single molecule diffraction limited imaging data and super-resolution microscopy aggregate morphological features for both subgroups.** Analysis for statistical test assumptions detailed in Supplementary Table 4

|  | **Subgroup 1** | | | | **Subgroup 2** | | | |
| --- | --- | --- | --- | --- | --- | --- | --- | --- |
|  | **Method** | **PD** | **Control** | **P** | **Method** | **PD** | **Control** | **P** |
| α-synuclein count | Median, Wilcoxon | 163.03 | 62.09 | 0.063 | Mean, students Ttest | 166.25 | 124.23 | 0.167 |
| Aβ count | Median, Wilcoxon | 202.25 | 214.16 | 0.74 | Median, Wilcoxon | 84.50 | 111.22 | 0.436 |
| α-synuclein/Aβ | Median, Wilcoxon | 1.072 | 0.48 | 0.029 | Mean, Welch Ttest | 2.18 | 1.17 | 0.031 |
| α-synuclein >0.02µm^2^ | Mean, Ttest | 0.071 | 0.066 | 0.62 | Mean, Ttest | 0.158 | 0.183 | 0.297 |
| Aβ >0.03µm^2^ | Mean, Ttest | 0.092 | 0.053 | 0.036 | Mean, Ttest | 0.099 | 0.055 | 0.0204 |
| α-synuclein circularity <0.4 | Mean, Ttest | 0.143 | 0.138 | 0.74 | Mean, Ttest | 0.077 | 0.105 | 0.265 |
| Aβ circularity <0.4 | Mean, Ttest | 0.14 | 0.09 | 0.025 | Mean, Ttest | 0.049 | 0.019 | 0.0152 |
| Combined discriminator | Mean, Ttest | 0.068 | 0.016 | 0.018 | Mean, Ttest | 0.097 | 0.024 | 0.0247 |

**Supplementary Table 4 Summary of count and morphology data tests for distribution (Shapiro-Wilks) and homogeneity of variance (Levene’s)**

|  | **Subgroup 1** | | | | | **Subgroup 2** | | | | |
| --- | --- | --- | --- | --- | --- | --- | --- | --- | --- | --- |
|  | **HC normality** | **PD normality** | **Levene’s** | **Test** | **p** | **HC normality** | **PD normality** | **Levene’s test** | **Test** | **p** |
| Age | 0.0350 | 0.737 |  | Wilcox | 0.0288 | 0.322 | 0.316 | 0.644 | Students | 0.0655 |
| α-synuclein count | 0.004741 | 0.00024 |  | Wilcox | 0.063 | 0.0888 | 0.0983 | 0.93 | Students | 0.167 |
| Aβ count | 0.56 | 0.00093 |  | Wilcox | 0.74 | 0.0672 | 0.0281 |  | Wilcox | 0.436 |
| Ratio | 0.02844 | 0.0027 |  | Wilcox | 0.029 | 0.7073 | 0.780 | 0.0439 | Welch | 0.031 |
| α-synuclein >0.2µm^2^ | 0.80 | 0.46 | 0.38 | Students | 0.62 | 0.524 | 0.333 | 0.0562 | Students | 0.297 |
| Aβ >0.03µm^2^ | 0.96 | 0.96 | 0.02 | Welch | 0.046 | 0.0742 | 0.323 | 0.0001 | Welch | 0.0204 |
| α-synuclein circularity <0.4 | 0.60 | 0.28 | 0.55 | Students | 0.75 | 0.119 | 0.076 | 0.528 | Student | 0.265 |
| Aβ circularity <0.4 | 0.071 | 0.16 | 0.08 | Students | 0.026 | 0.917 | 0.854 | 0.0208 | Welch | 0.0152 |
| Combined discriminator | 0.0616 | 0.243 | 0.0283 | Welch | 0.0185 | 0.874 | 0.877 | 0.0565 | Students | 0.0247 |

**Supplementary Table 5 Summary of super-resolution morphology features of aggregates from both subgroups obtained from dSTORM imaging**

|  | **Subgroup 1** | | **Subgroup 2** | |
| --- | --- | --- | --- | --- |
|  | **Mean** | **SD** | **Mean** | **SD** |
| PD α-synuclein area (µm^2^) | 0.012 | 0.042 | 0.013 | 0.023 |
| Control α-synuclein Area (µm^2^) | 0.012 | 0.056 | 0.013 | 0.018 |
| PD Aβ Area (µm^2^) | 0.013 | 0.031 | 0.014 | 0.031 |
| Control Aβ Area (µm^2^) | 0.011 | 0.073 | 0.011 | 0.011 |
|  | **Median** | **IQR** | **Median** | **IQR** |
| PD α-synuclein area (µm^2^) | 0.005 | 0.011 | 0.007 | 0.013 |
| Control α-synuclein Area (µm^2^) | 0.005 | 0.010 | 0.007 | 0.013 |
| PD Aβ Area (µm^2^) | 0.006 | 0.013 | 0.008 | 0.013 |
| Control Aβ Area (µm^2^) | 0.006 | 0.011 | 0.007 | 0.011 |
|  | **Mean** | **SD** | **Mean** | **SD** |
| PD α-synuclein circularity | 0.572 | 0.216 | 0.612 | 0.184 |
| Control α-synuclein circularity | 0.578 | 0.217 | 0.611 | 0.185 |
| PD Aβ circularity | 0.576 | 0.211 | 0.612 | 0.187 |
| Control Aβ circularity | 0.614 | 0.192 | 0.639 | 0.171 |

**Supplementary Table 6 Summary of total protein concentration measured using ELISA**

| **Protein** | **PD n** | **HC n** | **PD normality** | **HC normality** | **Levene’s** | **Test (statistic)** | **P** |
| --- | --- | --- | --- | --- | --- | --- | --- |
| α-synuclein | 15 | 16 | 0.116 | 0.531 | 0.412 | Students (24.174) | 0.660 |
| Aβ_40_ | 16 | 17 | 0.002 | <0.001 |  | Wilcox (103) | 0.242 |
| Aβ_42_ | 19 | 19 | <0.001 | <0.001 |  | Wilcox (201.5) | 0.549 |
| Aβ_42_/ Aβ_40_ | 16 | 17 | 0.206 | <0.001 |  | Wilcox (171) | 0.217 |

**Supplementary Table 7 summary of group comparisons for combined subgroups demographic, single molecule count data, and morphology data.**

| **Combined subgroups** | **HC normality** | **PD normality** | **Levene** | **Test (test statistic)** | **p** |
| --- | --- | --- | --- | --- | --- |
| Age | 0.895 | 0.966 | 0.0655 | Student’s (2.39) | 0.0229 |
| α-synuclein/Aβ aggregate count | 0.088 | 0.0249 |  | Wilcox (118) | 0.026 |
| Combined descriminator | 0.0172 | 0.0214 |  | Wilcox (41) | <0.001 |

**Supplementary Table 8 Summary of combined data from subgroups 1 and 2 with group wise comparison of combined discriminator values.**

|  | **PD** | **Control** | **P** |
| --- | --- | --- | --- |
| n | 18 | 17 |  |
| Age (years) mean ± SD | 64.16 ± 7.55 | 70.04 ± 6.98 | 0.0226 |
| Combined discriminator – median ± IQR | 0.07 ± 0.058 | 0.014 ± 0.025 | <0.001 |

**Supplementary Table 9 comparison of two logistic regression models using the combined data from subgroups 1 and 2.** Age has a non-significant effect when included in the model and when holding age constant the β-coefficient is only marginally decreased suggesting that the combined discriminator is the major independent variable impacting group prediction. This is further reflected in the Pseudo R^2^ values of each model which are close in value.

| $PD probability= \beta_{0}+ \beta_{1}\times Combined Descriminator$ | | | | |
| --- | --- | --- | --- | --- |
| **Model 1** | **β Coefficient** | **Standard Error** | **Z-value** | **P** |
| Combined discriminator | 63.2 | 23.6 | 2.67 | 0.00753 |
| Pseudo R^2^ =0.376 (p=1.96x10^-5^) | | | | |
| $PD probability= \beta_{0}+ \beta_{1}\times Combined Descriminator+ \beta_{2} \times age$ | | | | |
| **Model 2** | **β Coefficient** | **Standard Error** | **Z-value** | **P** |
| Combined Discriminator | 59.3 | 23.7 | 2.50 | 0.0123 |
| Age | -0,0760 | 0.0740 | -1.027 | 0.305 |
| Pseudo R^2^ = 0.398 (p=1.109x10^-5^) | | | | |
